# A Web-Based and Print-Based Computer-Tailored Physical Activity Intervention for Prostate and Colorectal Cancer Survivors: A Comparison of User Characteristics and Intervention Use

**DOI:** 10.2196/jmir.7838

**Published:** 2017-08-23

**Authors:** Rianne Henrica Johanna Golsteijn, Catherine Bolman, Denise Astrid Peels, Esmee Volders, Hein de Vries, Lilian Lechner

**Affiliations:** ^1^ Department of Psychology and Educational Sciences Open University of the Netherlands Heerlen Netherlands; ^2^ Department of Health Promotion Maastricht University Maastricht Netherlands

**Keywords:** eHealth, web-based intervention, print-delivered intervention, computer tailoring, intervention usage, physical activity, prostate cancer, colorectal cancer, cancer survivorship

## Abstract

**Background:**

Physical activity (PA) is beneficial in improving negative physical and psychological effects of cancer. The rapidly increasing number of cancer survivors, resulting from aging and improved cancer care, emphasizes the importance to develop and provide low cost, easy accessible PA programs. Such programs could be provided through the Internet, but that could result in the exclusion of cancer survivors not familiar with the Internet. Therefore, we developed a computer-tailored PA intervention for prostate and colorectal cancer survivors in which both Web-based and print materials are provided, and participants can choose their own preferred delivery mode.

**Objective:**

The aim of this study was to assess participants’ characteristics related to delivery mode and use of intervention materials.

**Methods:**

We studied characteristics of participants using Web-based and printed intervention materials in a randomized controlled trial (RCT). Prostate and colorectal cancer survivors recruited from hospitals were randomized to OncoActive (computer-tailored PA intervention) or a usual-care control group. OncoActive participants received both Web-based and printed materials. Participants were classified into initial print- or Web-based participants based on their preferred mode of completion of the first questionnaire, which was needed for the computer-tailored PA advice. Intervention material use during the remainder of the intervention was compared for initial print- or Web-based participants. Additionally, participants were classified into those using only print materials and those using Web-based materials. Differences in participant characteristics and intervention material use were studied through analysis of variance (ANOVAs), chi-square tests, and logistic regressions.

**Results:**

The majority of the participants in the intervention group were classified as initial Web-based participants (170/249, 68.3%), and 84.9% (191/249) used Web-based intervention materials. Dropout was low (15/249, 6.0%) and differed between initial Web-based (4/170, 2.4%) and print-based (11/79, 14%) participants. Participants were less likely to start Web-based with higher age (odds ratio [OR]=0.93), longer time since last treatment (OR=0.87), and higher fatigue (OR=0.96), and more likely with higher education (OR=4.08) and having completed treatments (OR=5.58). Those who were older (OR=0.93) and post treatment for a longer time (OR=0.86) were less likely to use Web-based intervention materials. Initial print-based participants predominantly used print-based materials, whereas initial Web-based participants used both print- and Web-based materials.

**Conclusions:**

To our knowledge, this is one of the first studies that assessed participant characteristics related to delivery mode in an intervention in which participants had a free choice of delivery modes. Use of print-based materials among the initial Web-based participants was substantial, indicating the importance of print-based materials. According to our findings, it may be important to offer Web- and print-based materials alongside each other. Providing Web-based materials only may exclude older, less educated, more fatigued, or currently treated participants; these groups are especially more vulnerable and could benefit most from PA interventions.

## Introduction

Cancer and cancer treatment coincide with short- and long-term effects on both physical and mental health, eventually decreasing quality of life of cancer patients and survivors (CPS) [1-6]. A healthy lifestyle, and especially physical activity (PA), is known to be beneficial for cancer survivors in improving treatment-related side effects and thereby health-related quality of life (HRQoL) [7-10]. Additionally, PA is a preventive factor for the development of other chronic diseases and comorbidities for which cancer survivors are at risk (eg, obesity, coronary heart disease, and diabetes), as well as for secondary or new cancer or cancer recurrence [10-15]. Therefore, effective PA programs for CPS are of major importance, especially since studies regarding supportive care needs have shown that CPS themselves express a substantial need for healthy lifestyle information and programs including PA [16-18].

In light of the rapidly growing population living with or after cancer, because of advances in early detection and treatment [19,20], there is a clear need for easily accessible and affordable programs aimed at self-management. Web-based interventions may be a cost-effective method since they have a large potential reach for low cost and have proven to be effective in increasing PA in both healthy and diseased populations [21-24]. A frequently used and proven effective method for Web-based interventions is computer-tailoring [23-26] where participants receive personalized feedback generated automatically using computer-based data-driven decision rules and data collected from questionnaires (eg, individual characteristics, beliefs, and behavior) [27].

With rapid increases in Internet access in recent years, preconditions for the use of Web-based interventions have improved substantially. In 2016, 94% of the Dutch population had Internet access and electronic health (eHealth) applications were increasingly used, especially by adults aged over 65 years and adults with a chronic disease [28,29]. Therefore, with a median age of 65 years at diagnosis [30], the use of eHealth for CPS seems promising. However, Internet access decreases substantially from the age of 75 years (60% compared with 90% among those aged 65-75 years in 2016), and frequency of Internet use is also substantially lower with increasing age [29]. eHealth literacy, that is, the ability to seek, find, understand, and appraise health information from electronic resources and apply that knowledge to solving a health problem or making a health-related decision [31], is important for eHealth interventions to be successful. Studies showed that older age and lower socioeconomic status (SES) are related to lower eHealth literacy [32] and that older adults may lack skills and knowledge for the use of eHealth interventions [33]. Interventions that are only provided through the Internet may therefore be less useful in a population of CPS (who are generally older aged) and may even exclude the elderly or those of lower SES from its benefits.

Alternatively, computer-tailored interventions can be delivered both through the Internet and in print. A Web-based version and a print-based version were offered alongside each other in the OncoActive intervention, a computer-tailored PA program to stimulate and maintain PA in prostate and colorectal CPS. As a result, CPS could choose their preferred delivery mode: every participant received log-in details for the OncoActive website to fill out the assessment questionnaire, as well as an additional (identical) paper-and-pencil version. After completion of the questionnaire of their own choice, participants received their tailored advice both Web-based and by normal mail, enabling them to use either one or both. Providing the ability to use the preferred method for accessing intervention materials can increase intervention reach and adherence and may eventually result in larger behavior change effects in the target population. Therefore, it is important to determine which participant characteristics (eg, demographics, disease related-factors, and health-related factors) are associated with the preference for a certain delivery mode and with the use of intervention materials. As providing the printed delivery mode alongside the Web-based intervention is associated with higher costs, it is also important to gain insight into the actual use of these materials.

Research relating participant characteristics to delivery mode preference is scarce. To our knowledge, there is only one study in which participants from a general adult population could freely choose between print-based and Web-based intervention materials. Factors associated with choosing printed materials were being older, less educated, and of poorer health status [34]. Another study examining participant characteristics of adults aged over 50 years cluster-randomized to either a print- or Web-based PA intervention found that there was a higher percentage of males in the Web-based intervention and that participants in the Web-based intervention were younger, had a higher body mass index (BMI), and a lower intention to be physically active [35]. A study from Short et al [36] regarding PA intervention preferences of the general adult population (comparing face-to-face, group-, print-, and Web-based delivery mode) revealed that factors positively associated with preference for a Web-based intervention were being middle aged, living in a rural area, and high Internet use. Web-based preference was negatively associated with female gender, obesity, and high PA participation. Preference for a print-based intervention was positively associated with older age and negatively associated with female gender and obesity [36]. A positive attitude toward eHealth interventions in a population of cancer survivors was associated with lower age, higher income, higher quality of life, having completed cancer treatment, and having prostate cancer [16].

The aim of this study was to provide insight into the characteristics of participants who initially chose to participate Web-based versus those who initially chose to participate in the print-delivered intervention. As participants could use both Web-based and printed materials or a combination after the initial choice, we also examined intervention material use and participant characteristics related to this. On the basis of findings in previous studies, we expected that age and education would be important predictors of initial Web-based participation and using Web-based intervention materials. Analyses with regard to PA and disease-related factors were exploratory.

Information regarding participant characteristics related to the initial choice for a delivery mode and the delivery mode and material use during the complete intervention would aid further implementation, as it could provide insight into the feasibility of using Web-based interventions in a population of CPS, which often is elderly. This information could also help future researchers to choose the appropriate delivery mode for their audience and provide insight in which persons may be hard to reach when providing only a Web-based intervention.

## Methods

### Study Design

This study is part of a randomized controlled trial (RCT) in which participants were randomized to either the OncoActive intervention group or a usual-care waiting-list control group to assess the effectiveness. Since this study only examines the intervention delivery mode, control group participants were excluded from the analyses. The RCT was approved by the Medical Ethics Committee of the Zuyderland hospital (NL47678.096.14) and is registered in the Dutch Trial Register (NTR4296). All participants provided written informed consent.

### Participants

CPS (≥18 years) diagnosed with colorectal or prostate cancer could participate in the trial if they were undergoing treatment with a curative intent or if they successfully completed primary treatment (surgery, chemotherapy, or radiation) up to 1 year ago. There was no restriction for patients currently undergoing hormonal therapy. By selecting only two cancer types, we could better fine-tune the intervention to the specific needs and capabilities in relation to cancer type. Prostate cancer and colorectal cancer were selected because they are among the most common cancer types in the Netherlands. Furthermore, survival rates are good, indicating a large population possibly benefiting from a PA intervention [30,37].

Participants should have had surgery at least 6 weeks before the start of the study. Those suffering from severe medical, psychiatric, or cognitive illnesses (eg, Alzheimer disease and mobility limitations) that could interfere with participation in a PA program were not invited to participate. Proficient Dutch reading and speaking skills were required for completing questionnaires and reading the tailored advice. Lack of Internet access and Internet skills were not a reason for exclusion.

### Procedure

Prostate and colorectal CPS were recruited from the urology or oncology departments of 17 hospitals in 2015 and 2016. Eligible CPS were identified by hospital staff, verbally informed (either in person or by telephone) about the study, and invited to participate. Written information was handed over or sent by mail if the patient agreed to receive an information package. Additionally, CPS were recruited via other channels (eg, calls in local newspapers, on relevant websites, discussion groups, and flyers in hospitals). Participants responding to these messages were informed by the researchers and were also sent an information package by mail.

The information package included a letter with information, a time schedule, an informed consent form, and a prepaid return envelope. If there was no response to the initial information package, 3 weeks later one postal reminder was sent. CPS who agreed to participate were randomized into either the intervention group or the control group. Subsequently, all participants wore an accelerometer (ActiGraph GT3X-BT, ActiGraph, Pensacola, FL) to objectively assess PA. Immediately after wearing the accelerometer for 7 days, every participant received an email with log-in details for the OncoActive website together with an invitation to fill out the Web-based questionnaire and an identical paper-and-pencil version of the questionnaire by normal mail, enabling them to fill out the version of their preference. After completing this baseline questionnaire (T0), the intervention group received the OncoActive intervention that is outlined below. Both groups had to fill out follow-up questionnaires at three time points: 3 (T1), 6 (T2), and 12 (T3) months after baseline. At each time point, participants could choose whether to fill out the Web-based questionnaire or the paper-based questionnaire. The T1 questionnaire was used to provide ipsative feedback in the form of tailored advice (see below). The questionnaires at 6 and 12 months were administered for efficacy and process evaluation purposes and were thus not considered part of the intervention. The T3 questionnaire is not part of this study.

### The OncoActive Intervention

The OncoActive intervention is a computer-tailored intervention aimed at awareness, initiation, and maintenance of PA behavior in prostate and colorectal CPS. The intervention was based on a proven effective evidence-based intervention to stimulate PA in adults over 50 years [38,39] and adapted for prostate and colorectal CPS using the intervention mapping protocol [40].

Participants in the intervention group received tailored PA advice at three time points. The content of the first and second tailored advice was based on information gathered with the baseline questionnaire. Both the baseline (T0) and the second questionnaire (T1) provided input for the third tailored advice and allowed for the provision of ipsative feedback. The content of the advice is based on behavior change theories and targets premotivational constructs (eg, awareness and knowledge), motivational constructs (eg, self-efficacy, attitude, and intrinsic motivation), and postmotivational constructs (eg, goal setting, action and coping planning, and self-regulation) [40-42]. In addition to the tailored advice, every participant received a pedometer and access to interactive content on the website (eg, role model videos, home exercise instruction videos, a module for goal setting using a pedometer, the option to consult a physical therapist, and additional information). A more detailed description of the intervention content can be found elsewhere [40], and some screenshots can be found in Multimedia Appendix 1.

As previously mentioned, every participant received the first questionnaire Web-based and on paper. After completion of the questionnaire of their own choice, participants received their tailored advice. If the questionnaire was completed on the website, advice was immediately available on the website, and participants were made aware that they would receive a printed version of their advice within 3 days. If participants completed the paper-and-pencil questionnaire, the advice text was available (Web-based and print-based) within 2 weeks after receiving the questionnaire, after uploading participant data by research staff. Participants were emailed (if they provided their email address) that their advice was available on the website and that they would receive a printed version of the advice within 3 days. The tailored text was exactly the same for both modalities, but the Web-based version contained more interactive content (eg, videos). All participants were made aware that they could find additional interactive content on the website. The tailored advice was displayed on a distinct section of the website.

For the second provision of advice (2 months after the start), participants received an email to notify them that their advice was available on the website and that they would receive a printed version within a few days. For the third provision of advice (within 2 weeks of completing the T1 questionnaire), participants again received 2 versions (Web and print) of a questionnaire, with a procedure similar to the first advice.

### Measurements

Several demographic variables, cancer-related characteristics, PA behavior, PA determinants, and health-related outcomes were measured in the baseline questionnaire of the RCT [40]. For this study, we used the following demographic variables: age, gender, height, weight, highest educational level, and household income. Educational level was categorized into low (ie, primary, basic vocational, or lower general school), moderate (ie, medium vocational school, higher general secondary education, and preparatory academic education), or high (ie, higher vocational school or university level) according to the Dutch educational system. Height and weight were used to calculate BMI (ie, weight in kilograms divided by height in meters squared). Participants were classified as being overweight (BMI >24.9 kg/m^2^) or not. Cancer-related characteristics included type of cancer, which was either prostate or colorectal in this study; treatment status; and date of their last treatment.

PA was measured in two ways. Self-reported PA was measured using the validated Short Questionnaire to Assess Health Enhancing Physical Activity (SQUASH) [43], assessing activities regarding commuting, household, occupation, and leisure time. Total minutes of PA were classified into light (metabolic equivalent [MET] <3.0), moderate (MET 3.0-5.9), and vigorous (MET >6) [44]. Minutes of moderate to vigorous PA (MVPA) were calculated by adding up total time in moderate and vigorous PA. The SQUASH questionnaire has shown to have reasonable reliability (ρ=.58) and validity against an accelerometer (ρ=.45) [43].

Additionally, objective PA was measured using the ActiGraph GT3X-BT. Participants wore the accelerometer on an elastic belt on their right hip for 7 days. Data were downloaded and analyzed using ActiLife software (ActiGraph, Pensacola, FL). Measurements were considered valid if there were at least 3 days with at least 10 hours of wear time [45-47]. Nonwear periods were excluded from the analyses and were identified according to Choi et al [48]: intervals of at least 90 consecutive min of zero counts with allowance of a maximum of 2 min of nonzero counts during a nonwear interval. MVPA was calculated using Freedson-VM cut-off points based on 60 s epochs [49].

Intention to be sufficiently physically active was assessed using a scale of three items (alpha=.91) on a 10-point scale (eg, “To what extent do you intend to be sufficiently physically active?”) [35,39]. The score of the three items was averaged, resulting in a total score ranging from 1 to 10, with a high score indicating a high intention to be physically active.

HRQoL was measured using the European Organization for Research and Treatment of Cancer Quality of Life Questionnaire-C30 (EORTC QLQ-C30) [50]. The questionnaire comprises several scales, with the global health status scale providing an overview of general quality of life. Global health status was measured with two items (alpha=.85) on a 7-point scale. Scores were converted to scores ranging from 0 to 100, with a high score indicating a high HRQoL.

Fatigue was assessed using the Checklist Individual Strength (CIS) [51]. The subjective fatigue subscale assesses the experience of fatigue of participants. The eight items (alpha=.89) of the subscale are scored on a scale from 1 to 7, resulting in a total score in the range of 8 to 56.

Intervention material use was assessed with two questions per advice specifically aimed at the tailored advice: “Did you read your advice on paper?” and “Did you read your advice on the website?”; participants could identify whether they read the advice “completely,” “partly,” or “not.”

### Statistical Analysis

#### Dropout Analysis

Multiple logistic regression was performed to determine whether participants’ characteristics were predictors of dropout during the intervention (ie, at the 3-month follow-up questionnaire). Choice for the initial delivery mode was added as a variable to identify if one of the groups was more likely to drop out of the intervention. All predictors were forced into the model simultaneously (method Enter in Statistical Package for the Social Sciences [SPSS]).

#### Initial Choice Intervention Delivery Mode

For the analysis regarding the choice of the initial intervention delivery mode, we analyzed data from all participants who completed the baseline questionnaire. Classification into groups for the initial preferred intervention delivery mode was based on the way participants chose to complete the baseline questionnaire. In the accompanying information letter, participants were informed that they would immediately receive their first PA advice on the website if they completed the baseline questionnaire (used for the tailored advice) through the Internet. Participants completing the first questionnaire on the website were therefore classified as “initial Web-based participants,” and participants completing the first questionnaire on paper were classified as “initial print-based participants.”

Descriptive statistics on demographic factors (ie, age, sex, educational level, and household income), cancer-related factors (ie, type of cancer, treatment phase, and time since last treatment), PA-related factors (ie, self-reported and objective PA behavior and intention to be physically active), and health-related factors (ie, BMI, HRQoL, and fatigue) were calculated for the complete intervention group and split for “initial Web-based participants” and “initial print-based participants.”

Univariate one-way analysis of variance (ANOVA) and chi-square tests were used to determine significant differences between both groups. Multiple logistic regression (Enter method) was performed to determine differences in participant characteristics for initial intervention delivery mode choice.

Both educational level and household income are regarded as indicators of SES. We decided to include only educational level in the logistic regression as a previous study showed that compared with household income, education was more consistently predictive of eHealth use [52].

#### Linking Delivery Preference to Intervention Use

Use of the different tailored advice texts was assessed with self-report questions. Chi-square tests were performed to determine differences between the “initial Web-based participants” and the “initial print-based participants” with regard to the use of tailored advice. Additionally, differences regarding mode of completion of the second questionnaire (T1), which was part of the intervention, were assessed.

#### Continued Intervention Use Delivery Mode

On the basis of self-report regarding the use of the three sets of advice, participants were classified as “exclusively print-based participants,” “participants who used both Web-based and print-based materials,” and “exclusively Web-based participants.” As there were only 2 participants classified as “exclusively Web-based participants,” we chose to dichotomize this classification into “exclusively print-based participants” and “participants using Web-based materials.”

Multiple logistic regression (Enter method) was performed to determine differences in participant characteristics between both groups.

All analyses were performed using SPSS version 22 (IBM Corp., Armonk, NY). To check for the influence of uneven groups in the multivariate logistic regression analyses, nonparametric bootstrapping with 5000 replications was applied.

## Results

### Dropout Analysis

Within the intervention group, 232 participants out of the 249 enrolled at baseline completed the second questionnaire and received their final advice. Two participants who did not complete the second questionnaire missed just one questionnaire, whereas 15 participants opted out of the study, resulting in a dropout rate of 6.0%. Although dropout was limited, logistic regression analyses revealed that initial print-based participants were more likely to drop out (odds ratio [OR] 4.32, 95% CI 1.15–16.25). Among the initial Web-based participants, the dropout rate was 2.4% (4/170), and among the initial print-based participants, the dropout rate was 14% (11/79).

### Participant Characteristics and Initial Choice Intervention Delivery Mode

**Figure 1:**
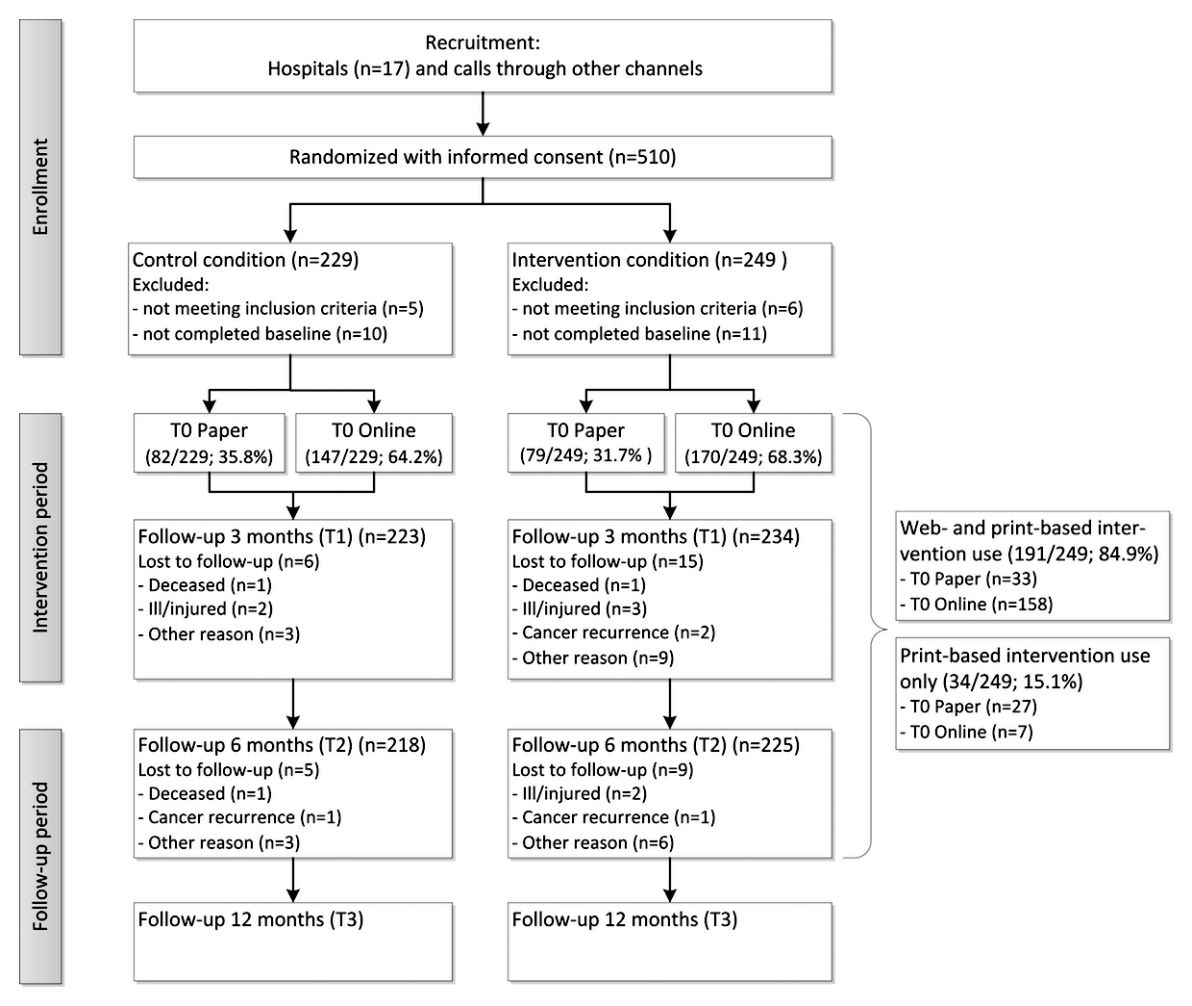
Flow diagram of the study. Note: the control condition was not included in this study.

In total, 510 prostate and colorectal CPS provided informed consent and were randomized into the intervention or the control group. For this study, we only used the data from the intervention condition, as this study aims to identify individual predictors of intervention delivery mode. In total, 249 participants were randomized into the intervention condition (Figure 1). Baseline characteristics for the complete intervention group are shown in Table 1.

The majority of the participants in the intervention group (n=249) were classified as initial Web-based participants (170/249, 68.3%). Significant differences between the initial Web-based participants and the initial print-based participants were found. Initial Web-based participants were significantly younger (*P*<.001) and higher educated (*P*=.002). Furthermore, initial Web-based participants had a higher income (*P*=.003), were more often post cancer treatment (*P*=.046), and were less fatigued (*P*=.04) (see Table 1).

**Table 1:** Baseline participant characteristics of the total intervention group and split for the initial Web-based participants and the initial print-based participants.

Characteristics			Total intervention (n=249)	Initial Web-based participants (n=170)	Initial print-based participants (n=79)	*P* value
**Demographic factors**						
	Age in years, mean (SD^a^)		66.38 (8.22)	65.08 (7.84)	69.18 (8.37)	<.001
	**Gender, n (%)**					.39
		Male	212 (85.1)	147 (86.5)	65 (82)	
		Female	37 (14.9)	23 (13.5)	14 (18)	
	**Education, n (%)**					.004
		Low	109 (44.0)	64 (37.6)	45 (58)	
		Middle	70 (28.2)	49 (28.8)	21 (27)	
		High	69 (27.8)	57 (33.5)	12 (15)	
	**Household income, n (%)**					.005
		Low	25 (12.8)	12 (8.1)	13 (23)	
		Middle	79 (38.5)	56 (37.6)	23 (41)	
		High	101 (49.3)	81 (54.4)	20 (36)	
**Cancer-related factors**						
	**Type of cancer, n (%)**					.08
		Prostate	149 (59.8)	108 (63.5)	41 (52)	
		Colorectal	100 (40.2)	62 (36.5)	38 (48)	
	**Treatment phase, n (%)**					.046
		During treatment	19 (7.6)	9 (5.3)	10 (13)	
		After treatment	230 (92.4)	161 (94.7)	69 (87)	
	Time since last treatment in months, mean (SD)		5.64 (3.84)	5.42 (3.65)	6.13 (4.22)	.18
**PA^b^-related factors**						
	MVPA^c^ SQUASH^d^, mean (SD)		798 (721)	831 (765)	727 (617)	.29
	MVPA ActiGraph, mean (SD)		270 (211)	280 (199)	249 (233)	.30
	PA intention, mean (SD)		7.61 (1.35)	7.71 (1.26)	7.38 (1.52)	.07
**Health-related factors**						
	**BMI^e^** **category, n (%)**					.47
		Normal weight	89 (36.2)	59 (34.7)	30 (40)	
		Overweight	157 (63.8)	111 (65.3)	46 (60)	
	General HRQoL^f^, mean (SD)		80.01 (16.81)	80.34 (16.53)	79.28 (17.51)	.65
	Fatigue, mean (SD)		24.01 (11.58)	23.04 (11.22)	26.48 (12.18)	.04

^a^SD: standard deviation.

^b^PA: physical activity.

^c^MVPA: moderate to vigorous physical activity.

^d^SQUASH: Short Questionnaire to Assess Health Enhancing Physical Activity.

^e^BMI: body mass index.

^f^HRQoL: health-related quality of life.

**Table 2:** Logistic regression to study relation between participant characteristics and the initial choice to participate Web-based (Nagelkerke *R*^2^=.25). Initial Web-based participation coded as 1. Additional nonparametric bootstrap analysis led to similar results.

Characteristics				Odds ratio (95% CI)		*P* value
**Demographic factors**						
	Age (years)		0.93 (0.89-0.98)		.005	
	Gender (male=Ref)		1.62 (0.52-5.03)		.40	
	**Education (low=Ref)**					
		Middle	0.95 (0.42-2.15)		.90	
		High	4.08 (1.58-10.56)		.004	
**Cancer-related factors**						
	Type of cancer (prostate=Ref)		0.55 (0.26-1.18)		.13	
	Treatment phase (during treatment=Ref)		5.58 (1.36-22.82)		.02	
	Time since last treatment (months)		0.87 (0.79-0.96)		.007	
**PA^a^-related factors**						
	PA intention		1.08 (0.80-1.44)		.62	
	MVPA^b^ ActiGraph		0.99 (1.0-1.0)		.33	
	Health-related factors					
	BMI^c^ (normal weight=Ref)		1.48 (0.73-3.03)		.28	
	General HRQoL^d^		1.00 (0.97-1.03)		.99	
	Fatigue		0.96 (0.93-1.00)		.04	

^a^PA: physical activity.

^b^MVPA: moderate to vigorous physical activity.

^c^BMI: body mass index.

^d^HRQoL: health-related quality of life.

Multiple logistic regression (see Table 2) revealed that participants were less likely to initially start Web-based with higher age (OR=0.93, 95% CI 0.89-0.98), longer time since last treatment (OR=0.87, 95% CI 0.79-0.96), and higher levels of fatigue (OR=0.96, 95% CI 0.93-1.0). Although time since last treatment is negatively associated with initially participating Web-based, participants who had completed cancer treatment were more likely to participate Web-based than those who were still under active treatment (OR=5.58, 95% CI 1.36-22.82). Furthermore, those with a high level of education were more likely to initially participate Web-based compared with those with a low level of education (OR=4.08, 95% CI 1.58-10.56).

### Linking Delivery Preference to Intervention Use

When examining intervention material use in relation to the initial choice for delivery mode (see Table 3), it can be noticed that a significantly higher percentage of initial print-based participants did not read (all three) Web-based advice (advice 1 and 2: *P*<.001; advice 3: *P*=.005). Furthermore, initial print-based participants were very consistent in their intervention material use throughout the intervention: 95% to 98% (partly) read the print-based advice and 56% to 62% did not read the Web-based advice (see Table 3). Web-based participants were more variable in the way they read their advice: completeness per advice decreases from the first advice to the final advice, with the final print-based advice being read significantly less completely (*P*<.001) by initial Web-based participants compared with initial print-based participants. Additional analyses showed that intervention completeness considering both versions of the advice was not lower for the initial Web-based participants. Percentages of participants reporting not having read any advice completely ranged from 0.9% (2/223) to 5.8% (13/225) per advice with no statistical differences between both groups.

With regard to completion of the second questionnaire (T1), it was noticed that the majority chose the same delivery mode for this questionnaire: 89.0% (146/164) of the initial Web-based participants completed the questionnaire on the website, and 86.8% (59/68) of the initial print-based participants completed the questionnaire on paper.

**Table 3:** Intervention material use and completeness compared for initial Web-based (n=170) and print-based (n=79) participants.

Reading of computer-tailored advice		Initial Web-based participants		Initial print-based participants		*P* value
		n	%	n	%	
**Advice 1 Print-based**						.36
	Completely	118	72.0	51	78	
	Partly	38	23.2	12	19	
	Not	8	4.9	1	2	
**Advice 1 Web-based**						<.001
	Completely	96	58.9	12	23	
	Partly	44	27.0	10	19	
	Not	23	14.1	31	59	
**Advice 2 Print-based**						.06
	Completely	102	62.2	50	77	
	Partly	39	23.8	12	19	
	Not	23	14.0	3	5	
**Advice 2 Web-based**						<.001
	Completely	83	51.2	11	21	
	Partly	40	24.7	9	17	
	Not	39	24.1	32	62	
**Advice 3 Print-based**						
	Completely	74	48.1	47	78	<.001
	Partly	58	37.7	10	17	
	Not	22	14.3	3	5	
**Advice 3 Web-based**						.005
	Completely	65	42.2	12	24	
	Partly	42	27.3	10	20	
	Not	47	30.5	28	56	

**Table 4:** Logistic regression to study relation between participant characteristics and the continued use of Web-based intervention materials (Nagelkerke *R*^2^=.21). Use of Web-based materials coded as 1. Additional nonparametric bootstrap analysis led to similar results.

Characteristics			Odds ratio (95% CI)		*P* value	
**Demographic factors**						
	Age (years)			0.93 (0.86-1.0)		.04
	Gender (male=Ref)			0.82 (0.18-3.63)		.79
	**Education (low=Ref)**					
		Middle		1.22 (0.39-3.79)		.73
		High		3.52 (0.98-12.61)		.05
**Cancer-related factors**						
	Type of cancer (prostate = Ref)			0.98 (0.33-2.87)		.96
	Treatment phase (during treatment = Ref)			0.99 (0.08-12.77)		.99
	Time since last treatment (months)			0.86 (0.75-0.98)		.02
**PA^a^-related factors**						
	PA intention			1.13 (0.78-1.65)		.51
	MVPA^b^ ActiGraph			1.00 (1.0-1.0)		.53
**Health-related factors**						
	BMI^c^ (normal weight=Ref)			2.34 (0.92-5.95)		.08
	General HRQoL^d^			1.00 (0.97-1.04)		.76
	Fatigue			0.98 (0.93-1.03)		.43

^a^PA: physical activity.

^b^MVPA: moderate to vigorous physical activity.

^c^BMI: body mass index.

^d^HRQoL: health-related quality of life.

### Continued Intervention Use Delivery Mode

With regard to the selected delivery mode for using the intervention materials, we noticed that the majority (191/225; 84.9%) used Web-based most often in combination with print-based materials (see Figure 1). Results of the logistic regression identifying participant characteristics concerning the delivery mode for the use of intervention materials (print-only vs using Web-based materials; see Table 4) were similar to the results for initial choice of delivery mode for age (OR=0.93, 95% CI 0.86-1.00) and time since last treatment (OR=0.86, 95% CI 0.75-0.98). Highly educated participants were not significantly more likely to use Web-based intervention materials than less educated participants, but the OR (3.52) and its 95% CI (0.98-12.61) indicate that educational level may still be an important predictor. Fatigue and treatment phase were not identified as predictors for using Web-based materials (or a combination) instead of using only print-based materials.

## Discussion

This study was aimed at investigating participant characteristics in relation to initial choice of intervention delivery mode in a population of prostate and colorectal CPS. Additionally, intervention material use and participant characteristics in relation to intervention delivery mode were examined. Analyses provide insight into the feasibility of Web-based interventions in an older population of cancer patients and thereby aid further implementation of the intervention.

### Participant Characteristics and Delivery Mode

Age and education level were two participant characteristics which were consistently related to intervention delivery mode both for initial choice and follow-up delivery mode (ie, Web-based vs print-based). Higher age was associated with a lower likelihood of using Web-based intervention materials. A lower educational level, although not significant in all analyses, was also associated with lower Web-based participation. This corresponds with our expectations. Previous studies also revealed that eHealth literacy is lower for older adults and those with lower education [33,53]. Kontos et al [52] found younger age and higher education to be predictors for searching health information through the Internet and using websites for diet, weight, and PA. In addition, participants in an adult population selecting print-based materials were older and had a lower level of education than those selecting Web-based materials [34].

This finding may imply that when implementing Web-based interventions in a population of prostate and colorectal CPS, but probably also in a general older population (ie, 61% of colorectal and 64% of prostate cancer patients is aged over 70 years at the time of diagnosis [30]), those who are older and those with a lower level of education may not be reached. Statistics in the Netherlands showed that Internet access and frequency of Internet use decrease substantially from the age of 75 years and are also lower among those with a lower educational level [29]. Thus, besides lower Internet access, older and less educated participants may also have lower Internet experience and self-efficacy. As a result, they may choose to use the print-based materials, requiring less effort in comparison with the Web-based materials. This is acknowledged through the unified theory of acceptance and use of technology (UTAUT) by Venkatesh. This theory states that use of technology is influenced by (among others) facilitating conditions and performance and effort expectancy, which are moderated by age and experience [54]. Additionally, a study among colorectal CPS revealed that older patients do perceive Web-based health information tools as highly useful and indicate a willingness to use such tools but are not always able to use them optimally [55]. It may be recommended to provide both Web-based and print-based materials, especially among those aged over 75 years, to prevent exclusion of a vulnerable group of older or less educated participants and to have the most optimal use of the intervention

Besides age and education, time since treatment was also consistently related to participating in the Web-based intervention. CPS who finished their treatment longer ago were less likely to participate through the Internet. CPS who received their most recent treatment longer ago are probably already further in their recovery process, may perceive less need for PA advice and may be less committed to becoming physically active. As a result, they probably chose the delivery mode for which they needed the least effort. Using print-based materials may be perceived as easier, as all materials are delivered at home and can be completed at any time. Although accessing Web-based materials was made as easy as possible (eg, emails linked participants to the website without log-in), for Web-based participation, it is still necessary to start up a computer or tablet and go on the Web before being able to complete questionnaires [56]. Our results suggest that it may be important to provide print-based materials to also include those who completed their treatment longer ago. However, no other studies considered time since treatment, and therefore, additional research is necessary to explore the role of time since treatment.

Having finished treatment and lower levels of fatigue predicted the initial choice to participate Web-based but did not predict the use of Web-based intervention materials. Possibly, participants’ Internet frequency decreases during treatments and while feeling fatigued. Going on the Web to start the intervention may be perceived as more effortful and may explain the initial choice to participate in the print-based intervention. During the course of the intervention, treatments may be finished and fatigue may decrease. As a result, CPS may decide to visit the Web-based content of the intervention during continued use. It may be important to provide both delivery modes at invitation for those who are still undergoing treatment or suffering from fatigue, a group that may benefit most from the intervention. Future research needs to confirm these findings.

In this study, gender was not predictive for intervention delivery mode. The precise role of gender differences regarding Internet access, eHealth use, and delivery mode has been ambiguous: whereas some studies found a link with gender [35,36,52], others did not find differences between males and females [32,34,57,58]. Additionally, it should be noted that there was only a small portion of women in this study, as a result of part of the intended target population being prostate CPS, which may have influenced the power to detect differences. Future research should provide more insight regarding the influence of gender on delivery mode.

It is also interesting that PA behavior and intention to be physically active are not related to intervention delivery mode preference. PA behavior has proven to be a predictor of delivery mode preference according to studies examining self-reported intervention modality preference. Studies in the general population and in a cancer population found that those with lower PA levels may have a preference for Web-based or computer-based interventions [36,57]. Others argued that those with a risk behavior (eg, low PA behavior) may prefer the instant availability and interactivity of Web-based materials [34]. However, both in this study as well as in the study of Greaney et al [34], the actual choice of delivery mode was not predicted by PA behavior. Possibly, reporting a certain preference is different from the actual choice. Therefore, additional research is necessary to examine the role of health behavior in intervention delivery mode.

### Intervention Material Use

It is promising that in an older population (ie, mean age of 66 years), approximately two-thirds of the participants initially chose to participate through the Internet and that even a larger proportion (ie, almost 85%, 191/225) used the Web-based intervention content. This indicates that for a large part of our population, going on the Web was not a barrier.

Since the Web-based content of the OncoActive intervention could be accessed using a computer or tablet, participants were able to visit the website in the manner they were most familiar with (eg, computer or tablet). Providing OncoActive through different platforms may have increased the usage of the Web-based intervention materials [59].

We also examined whether intervention material use differed between initial print-based participants and initial Web-based participants. The majority of initial print-based participants predominantly used the print-based tailored advice. Significantly less participants in this group used the Web-based advice (ie, 38%-44%, Table 3), compared with the initial Web-based participants (ie, 69.5%-85.9%, Table 3). Additionally, the majority (ie, 87%, 59/68) of the initial print-based participants also completed the second questionnaire (which was part of the intervention) on paper. These findings indicate that it may be important to provide print-based intervention materials for participants who start the intervention print-based. However, since Web-based materials can be provided without additional costs, it is recommended to provide both.

The majority of the initial Web-based participants also completed the second questionnaire on the website (ie, 89.0%, 146/164) and used both Web-based (ie, 69.5%-85.9%, Table 3) and print-based (ie, 85.8%-95.2%, Table 3) intervention materials, indicating that initial Web-based participants used a mixture of both advice texts and that providing print-based tailored advice in addition to the Web-based advice may be advantageous. Studies among elderly found similar results, indicating that even among those using the Internet, a preference for print-based or nondigital information persists [33,60]. Although this may be a temporary phenomenon (eg, rapid technology development and aging of adults more familiar with the Internet), Vandelanotte et al [24] also suggested that having access to Web-based material might not be sufficient in itself. Therefore, future research should also focus on reasons for not using Web-based materials or why there is a preference for print-based materials.

As mentioned, information regarding use of print-based materials is important, as offering Web-based and print-based materials alongside each other is associated with higher costs. A version that is only Web-based would be less costly. With regard to the questionnaires, it was noticed that a majority started the intervention with a Web-based questionnaire and also continued to complete additional questionnaires on the website. Consequently, it may be feasible to initially invite participants to complete the questionnaires on the website, while explicitly explaining that it is also possible to participate in the intervention if they do not have Internet access or are not able or willing to participate through the Internet. Print-based questionnaires can then be provided on request or with a reminder. Nevertheless, it may be advisable to offer both delivery modes with the invitation for those who are older or for those still undergoing treatment, as these participant characteristics are easy to administer at intake, and results of this study showed that they are predictors of initial print-based participation.

With regard to the computer-tailored advice, the majority used a combination of both Web-based and printed materials. Although providing print-based materials complementary to the Web-based advice is associated with higher costs (eg, printing and postage costs), it may be best to provide both, as print materials are used by all participants. Providing both delivery modes alongside each other may be more costly than providing the intervention in a singular delivery mode. Additionally, intervention efficacy in relation to delivery mode should also be considered, as information processing may depend on the delivery mode [61]. Therefore, future research should also focus on the relation between delivery mode and (cost) effectiveness.

### Strengths and Limitations of This Study

Providing participants with the ability to select their own preferred intervention delivery mode is regarded as a strength of this study. As indicated by previous studies, this may enlarge intervention engagement and thereby the impact of the intervention [34]. Additionally, this study had a very low dropout rate. Only 6.0% (15/249) of the participants opted out of the study during the intervention period. This is a remarkable finding, as high dropout rates are common in Web-based interventions [62,63]. Providing a combination of Web-based and print-based materials might have prevented participants from dropping out of the study. If a specific delivery mode did not meet participants’ expectations, they were able to use only the materials that were most appealing to them.

As described in the methods, the preferred initial delivery mode was based on completion of the first questionnaire, as it contained the questions to build the tailored advice. However, it should be acknowledged that because of the evaluation of the intervention in an RCT, the questionnaire was longer than the actual questionnaire needed for tailoring. As a result, the current findings may reflect the preference for completing a research survey rather than the actual intervention delivery mode. Future implementation without the research component would be necessary to confirm the current findings.

In this study, we did not collect any information regarding Internet access and previous experience with the Internet. This information might have been valuable, as other studies found those factors to be predictors of using Web-based intervention materials [33,34,52].

Participants in this study were offered both Web-based and print-based materials complementary to each other. Therefore, we were not able to discriminate whether offering only one delivery mode would yield the same results. This could be studied in future research.

In our study, recall bias was possible. The use of tailored advice was self-reported, and evaluation took place up to 3 months after participants received advice. For future studies, it is recommended to incorporate some evaluation regarding the use of Web-based material immediately after providing the materials and preferably objective usage data. Objective usage data is not available in a print-based version and incorporating an additional questionnaire immediately after provision is more complicated and burdensome for the print-based material. However, objective usage data for Web-based material can be used to validate self-report items to assess the probability of recall bias in future studies.

### Conclusions

Intervention reach may be better, and interventions may possibly even be more effective if participants are able to use their own preferred delivery mode [34]. Information regarding participant characteristics related to intervention delivery mode can provide important cues for implementing computer-tailored interventions. To our knowledge, this is one of the first studies that assessed the relationship between participant characteristics and choice of delivery mode in an intervention in which both delivery modes were offered alongside each other, thereby providing participants a free choice of delivery mode.

Use of print-based materials among the initial Web-based participants was substantial, indicating that print-based materials are also important for those using Web-based materials. In contrast, by using only print-based materials, the intervention may be less attractive and useful for younger CPS, as it is known that younger CPS frequently use the Internet with regard to finding health-related information [64]. This study provides indications that Web-based and print-based materials could best be offered alongside each other. Providing Web-based materials only would exclude some of those who are older, less educated, more fatigued, or are currently undergoing treatment. Especially these participants are often more vulnerable and could benefit most from PA interventions.

## Appendix

Multimedia Appendix 1 Screenshots of the OncoActive intervention.

## Abbreviations

ANOVA: analysis of variance
BMI: body mass index
CIS: Checklist Individual Strength
CPS: cancer patients and survivors
eHealth: electronic health
EORTC QLQ-C30: European Organization for Research and Treatment of Cancer Quality of Life Questionnaire-C30
HRQoL: health-related quality of life
MET: metabolic equivalent
MVPA: moderate to vigorous physical activity
OR: odds ratio
PA: physical activity
RCT: randomized controlled trial
SD: standard deviation
SES: socioeconomic status
SPSS: Statistical Package for the Social Sciences
SQUASH: Short Questionnaire to Assess Health enhancing physical activity
UTAUT: Unified Theory of Acceptance and Use of Technology
