# Supplementary material for: A Web-Based and Print-Based Computer-Tailored Physical Activity Intervention for Prostate and Colorectal Cancer Survivors: A Comparison of User Characteristics and Intervention Use
Source: J Med Internet Res. 2017 Aug 23;19(8):e298. doi: 10.2196/jmir.7838 (PMC5587888; doi:10.2196/jmir.7838)
Supplement: Multimedia Appendix 1 [file jmir_v19i8e298_app1.pdf]

OncoActive

Tailored PA advice for prostate and colorectal cancer patients

Rianne Golsteijn
Catherine Bolman
Denise Peels
Esmee Volders
Hein de Vries
Lilian Lechner

OncoActive

- Computer-tailored PA intervention for prostate and colorectal cancer patients (during and after treatment)
- Aimed at:
  - creating awareness of own PA behavior
  - stimulating PA
  - maintaining PA behavior
- By targeting psychosocial determinants of PA behavior and integrating PA into daily life

Structure OncoActive

- Three times tailored advice, print- or Web-based

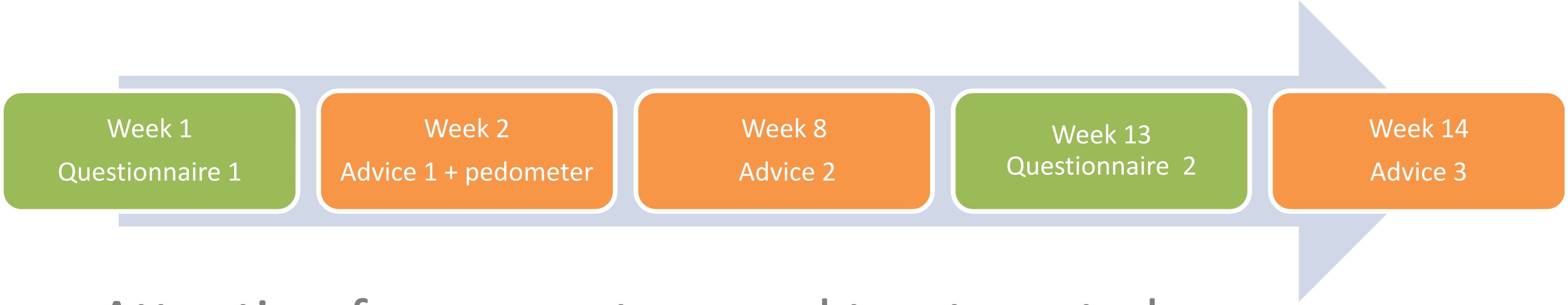

- Attention for cancer type and treatment phase
- A website with additional information and interactive content
- Pedometer

Screenshots Website

A A A

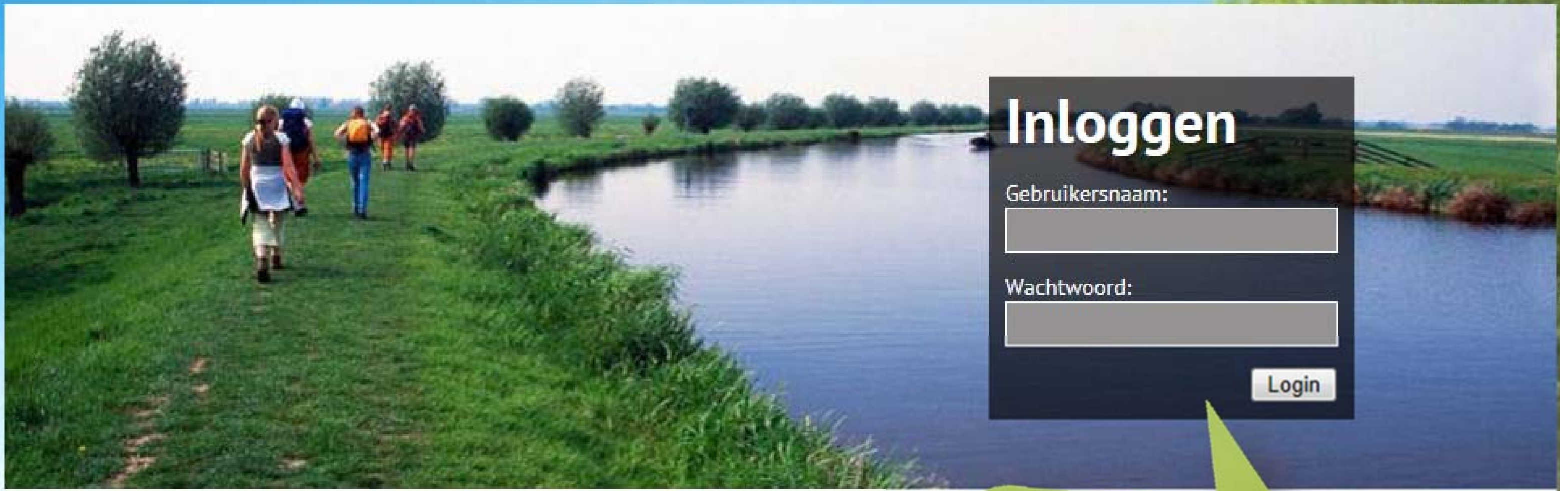

participants can log into the website

OncoActiefPlus

Wat is het en voor wie is het bedoeld?

OncoActiefPlus is een online informatie- en ondersteuningsprogramma voor mensen bij wie de primaire behandeling succesvol is afgerond. Het programma is gericht is op herstel na kanker. Gelijktijdig is er een onderzoek aan gekoppeld om te beoordelen wat de gebruikers van het programma vinden en of het hen helpt. Het project wordt uitgevoerd door de Open Universiteit in samenwerking met de Universiteit Maastricht. Het project wordt gefinancierd door KWF Kankerbestrijding.

OncoActiefPlus is alleen toegankelijk voor personen die hiervoor een uitnodiging ontvangen. Gegevens van deelnemers zijn zorgvuldig afgeschermd en worden veilig bewaard. Alleen de onderzoekers hebben toegang tot deze gegevens.

Hoe is het pr[illegible]

Deelnemers [illegible]
in. Een deel [illegible]
eerste vrage[illegible]
krijgen na e[illegible]
kunnen we b[illegible]

Wachtwoord vergeten?

Neem contact op met het OncoActiefPlus team (info@oncoactiefplus.nl).

NIEUWS

In november 2013 gaat het project van start. Tot en met juni 2014 kunnen nieuwe deelnemers instromen. Er zullen ongeveer 450 voormalige patiënten deelnemen. In 2015 worden de eerste resultaten verwacht.

Maastricht University   Open Universiteit   KWF Kanker Bestrijding

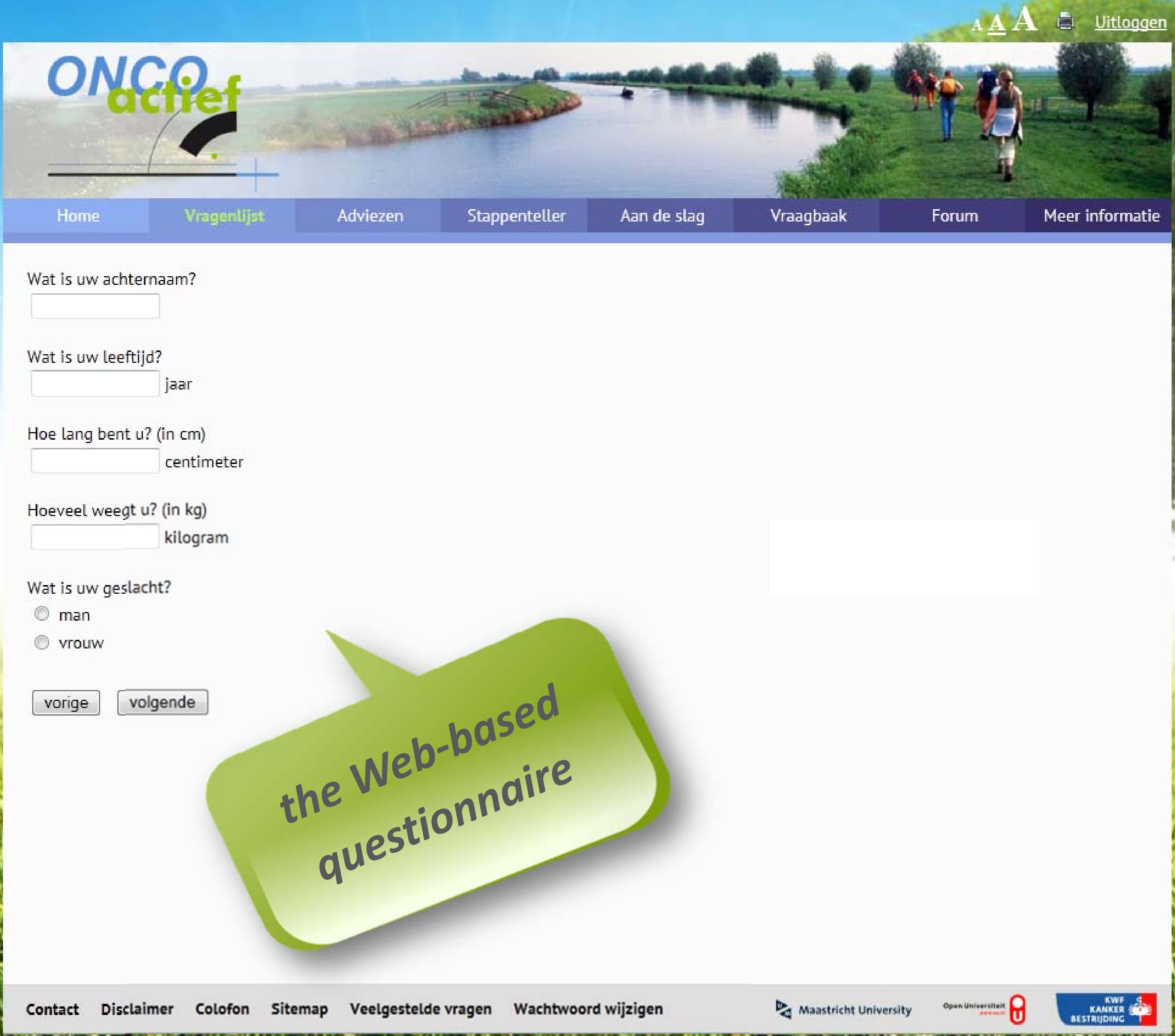
Uitloggen
ONCO actief
Home
Vragenlijst
Adviezen
Stappenteller
Aan de slag
Vraagbaak
Forum
Meer informatie
Wat is uw achternaam?
Wat is uw leeftijd?
jaar
Hoe lang bent u? (in cm)
centimeter
Hoeveel weegt u? (in kg)
kilogram
Wat is uw geslacht?
man
vrouw
vorige
volgende
the Web-based questionnaire
Contact
Disclaimer
Colofon
Sitemap
Veelgestelde vragen
Wachtwoord wijzigen
Maastricht University
Open Universiteit
KWF KANKER BESTRIJDING

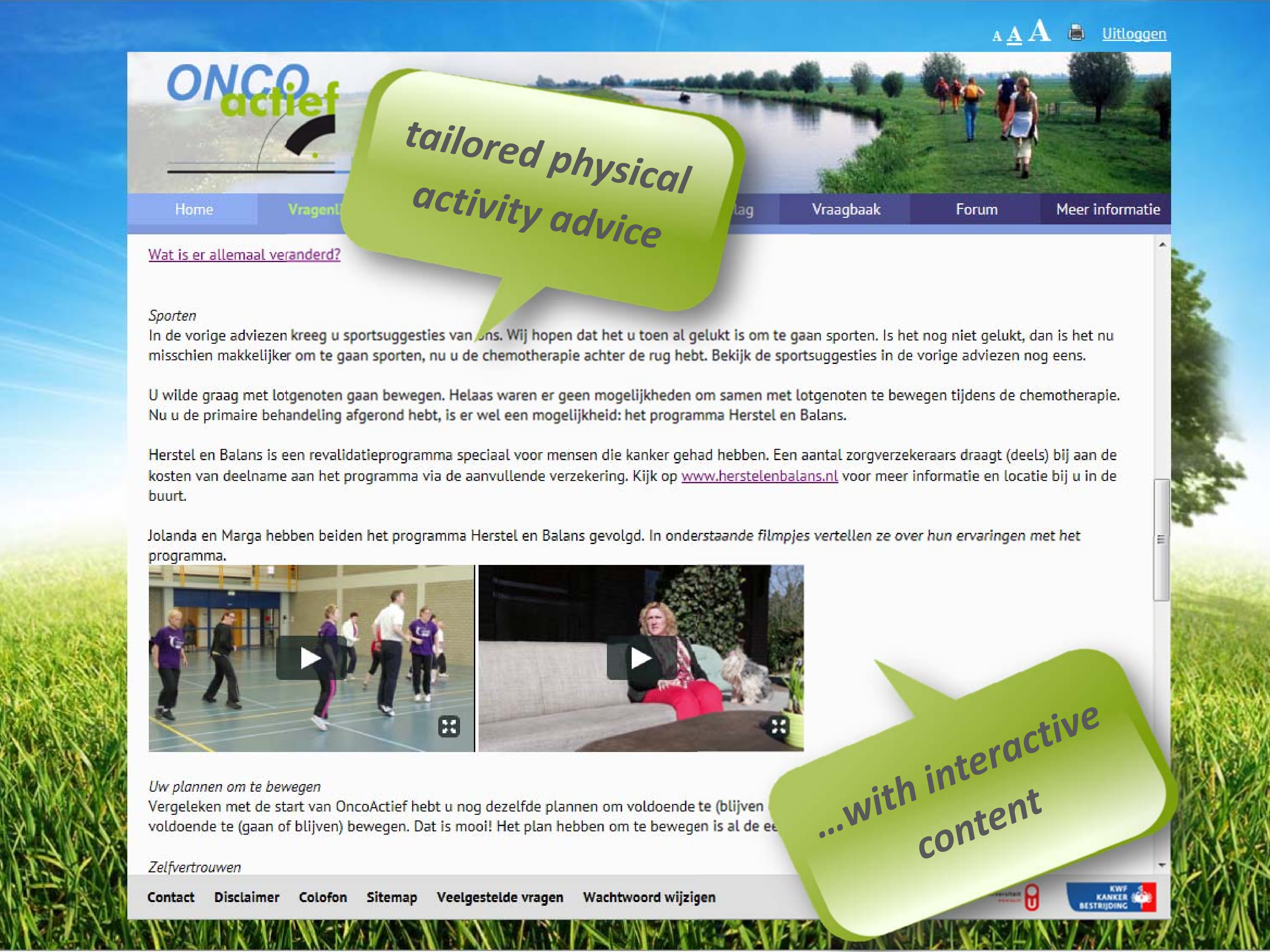
tailored physical activity advice
...with interactive content
A A A Uitloggen
ONCO actief
Home
Vraagbaak
Forum
Meer informatie
Wat is er allemaal veranderd?
Sporten
In de vorige adviezen kreeg u sportsuggesties van ons. Wij hopen dat het u toen al gelukt is om te gaan sporten. Is het nog niet gelukt, dan is het nu misschien makkelijker om te gaan sporten, nu u de chemotherapie achter de rug hebt. Bekijk de sportsuggesties in de vorige adviezen nog eens.
U wilde graag met lotgenoten gaan bewegen. Helaas waren er geen mogelijkheden om samen met lotgenoten te bewegen tijdens de chemotherapie. Nu u de primaire behandeling afgerond hebt, is er wel een mogelijkheid: het programma Herstel en Balans.
Herstel en Balans is een revalidatieprogramma speciaal voor mensen die kanker gehad hebben. Een aantal zorgverzekeraars draagt (deels) bij aan de kosten van deelname aan het programma via de aanvullende verzekering. Kijk op www.herstelenbalans.nl voor meer informatie en locatie bij u in de buurt.
Jolanda en Marga hebben beiden het programma Herstel en Balans gevolgd. In onderstaande filmpjes vertellen ze over hun ervaringen met het programma.
Uw plannen om te bewegen
Vergeleken met de start van OncoActief hebt u nog dezelfde plannen om voldoende te (blijven
voldoende te (gaan of blijven) bewegen. Dat is mooi! Het plan hebben om te bewegen is al de ee
Zelfvertrouwen
Contact
Disclaimer
Colofon
Sitemap
Veelgestelde vragen
Wachtwoord wijzigen
KWF KANKER BESTRIJDING

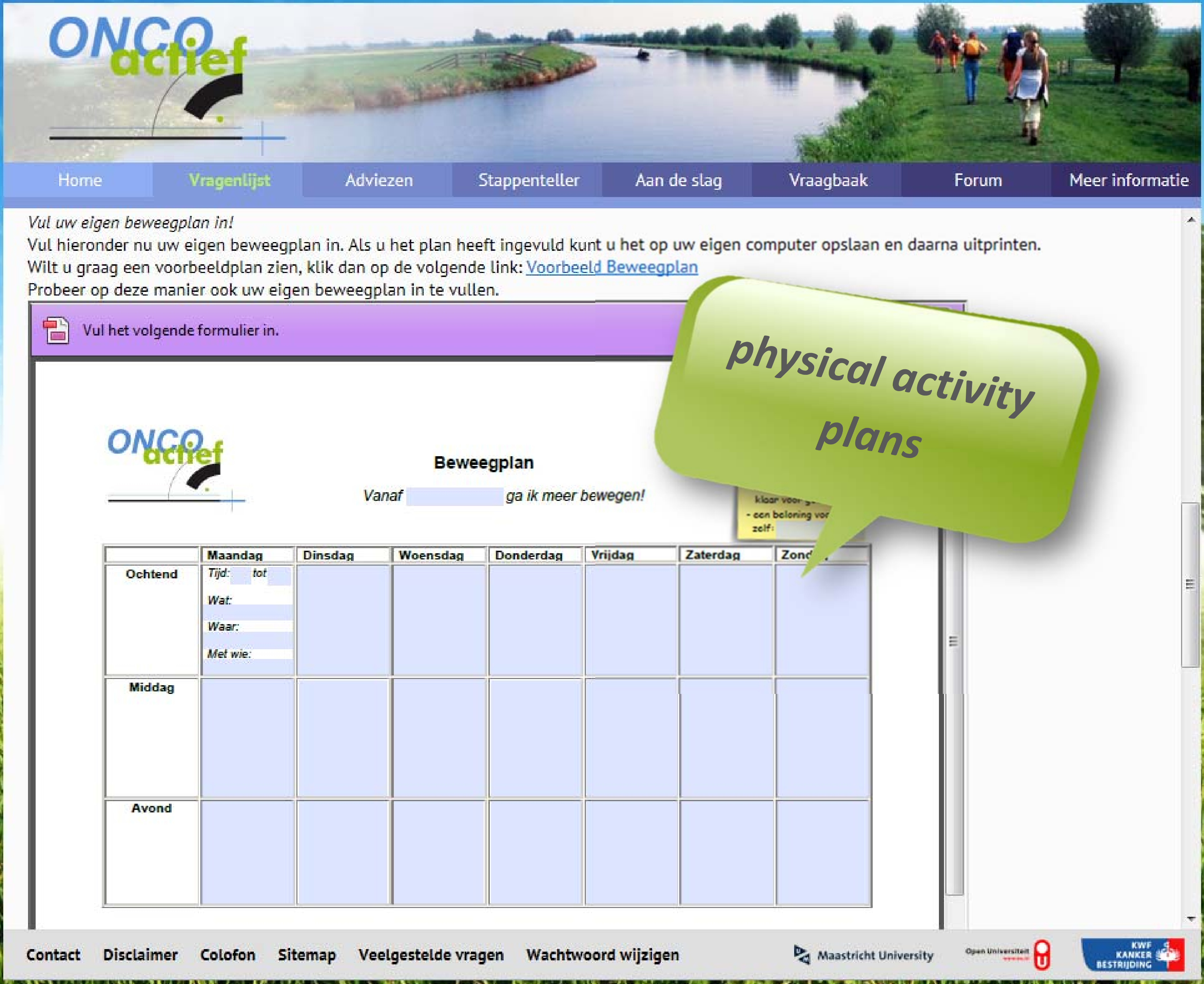

physical activity plans

A A A Uitloggen

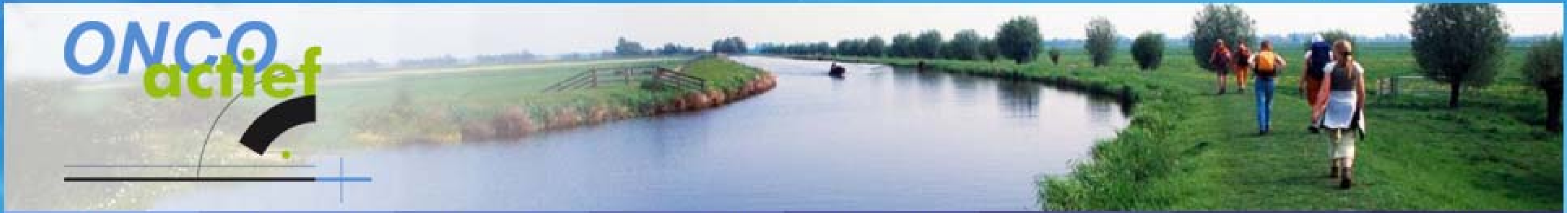

Home | Vragenlijst | Adviezen | Stappenteller | Aan de slag | Vraagbaak | Forum | Meer informatie

Beweegoefeningen

exercises to do at home

Beweegoefeningen

Hoewel u voor het behoud van uw gezondheid al voldoende beweegt, kan iets meer bew [illegible] Graag geven wij u dan ook twee beweegoefeningen die u naast u huidige beweegactivit [illegible] uis uitvoeren, en u bent met beide oefeningen binnen 5 minuten klaar. Kleine moeite dus, [illegible] n van deze oefeningen graag nog meer oefeningen wilt doen, raden wij u aan om 's ocht [illegible] nd 1 van 6.45-6.59u en van 9.15-9.30u. In het volgende advies, zult u weer enkele oefenin [illegible]

Deze eerste oefening versterkt uw kuiten en enkels en verbetert uw stabiliteit en balan [illegible]

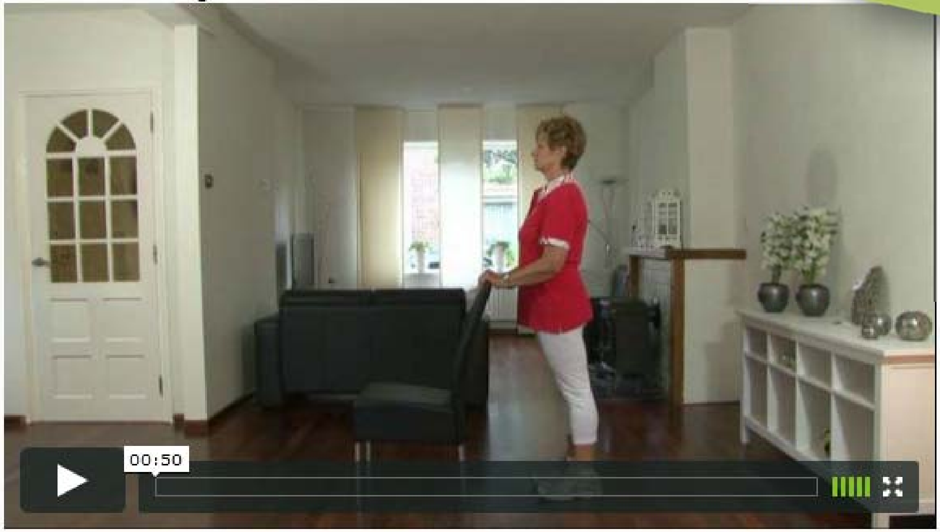

De volgende oefening versterkt uw armspieren, en is goed voor de spieren van uw borstkast.

Bij deze oefening wordt gebruik gemaakt van gewichtjes. Hiervoor kunt u gewoon een twee voorwerpen met een gelijk gewicht uit uw huis nemen die [illegible]

Contact | Disclaimer | Colofon | Sitemap | Veelgestelde vragen | Wachtwoord wijzigen

Maastricht University | Open Universiteit | KWF Kanker Bestrijding

A A A Uitloggen

ONCO actief

Home | Vragenlijst | Adviezen | Stappenteller | Aan de slag | Vraagbaak | Forum | Meer informatie

Welkom!

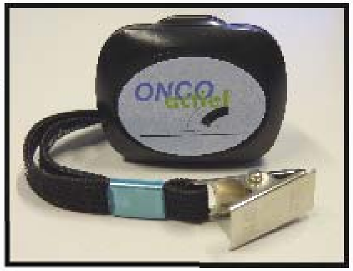

Welkom bij de Stappenteller Module! Wij hopen dat de stappenteller en het bijbehorende beweegadvies u kunnen helpen om meer te bewegen en actiever te leven. In deze module vindt u informatie over:

- De juiste manier om stappenteller te dragen.
- Het berekenen van uw stappendoel.

Bezoekt u voor de eerste keer de stappenteller module?

- Ja
- Nee

volgende

a pedometer to set PA goals

Contact | Disclaimer | Colofon | Sitemap | Veelgestelde vragen | Wachtwoord wijzigen

Maastricht University | Open Universiteit | KWF Kanker Bestrijding

Printed Intervention Materials

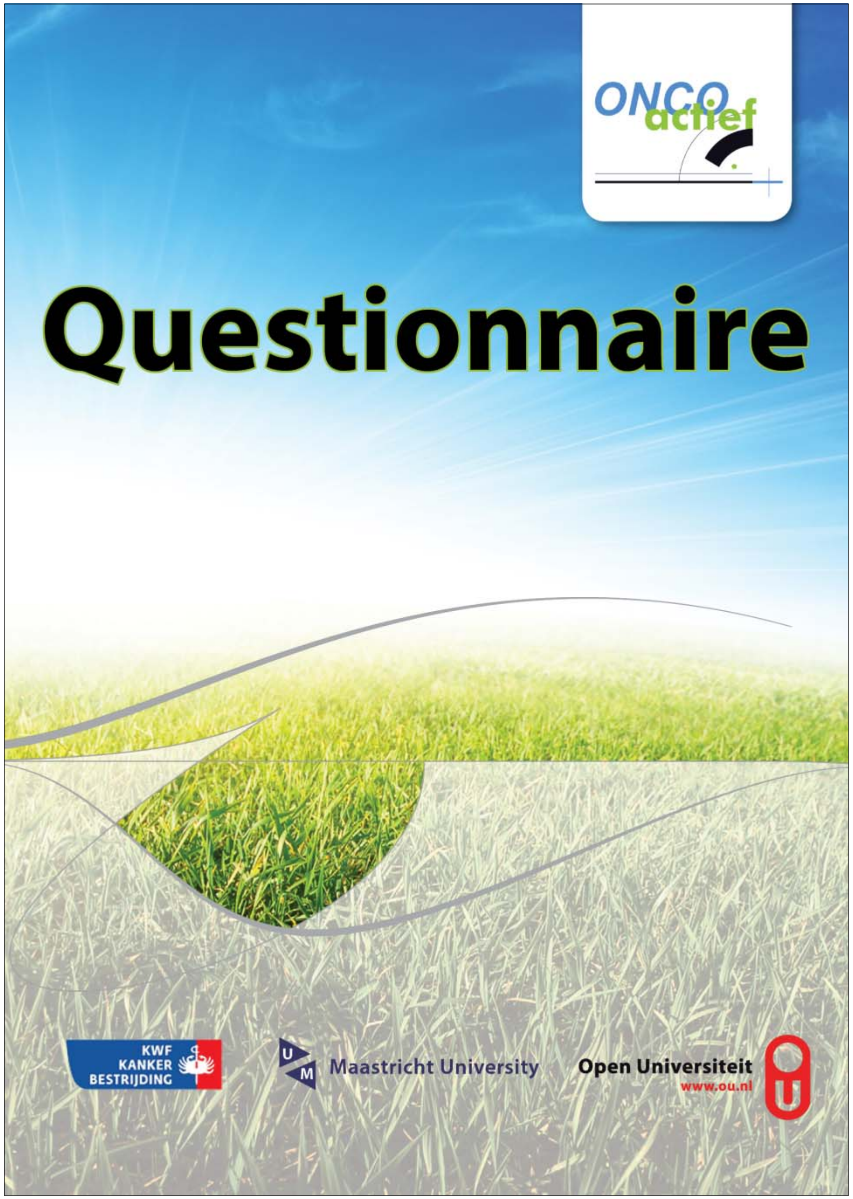

Persoonlijke gegevens

Wat is uw achternaam? ....

Wat is uw leeftijd? .... jaar

Hoe lang bent u? .... cm

Hoeveel weegt u? .... kg

Wat is uw geslacht? ☐ man ☐ vrouw

Wat is uw burgerlijke staat?

☐ gehuwd of samenwonend ☐ alleenstaand, gescheiden, ongehuwd of weduwe/weduwnaar

Wat is de hoogste schoolopleiding die u met een diploma hebt afgerond?

☐ lagere school/basisonderwijs
☐ lager algemeen of lager beroeps onderwijs (lbo, lts, leao, enzovoorts)
☐ middelbaar algemeen onderwijs ((m)ulo, mavo, mms)
☐ middelbaar beroeps onderwijs (mbo, mts, meao)
☐ hoger algemeen onderwijs (havo, hbs, vwo)
☐ hoger beroeps onderwijs (hts, heao, hbo, pabo, enzovoorts)
☐ wetenschappelijk onderwijs (universiteit)

Gezondheidssituatie

Bij het aanmelden voor het onderzoek hee[illegible] doorgegeven [illegible] deling u heeft gehad of momenteel nog steed[illegible] kanker. Daarom vragen we dit nu niet nog een [illegible]

Denkt u dat u over 2 maanden nog [illegible] bestraling) ondergaat?

☐ Ja, namelijk ....[illegible]
☐ Nee

Moet u op korte termijn[illegible]

☐ Ja, waarschijnlijk bi[illegible]
☐ Ja, maar pas over me[illegible]
☐ Nee

Soms kan de kanker ook uitge[illegible] in de buurt van de tumor, naar botten, of naar ande[illegible] zijn er bij u uitzaaiingen geconstateerd?

☐ Ja, namelijk in...
☐ Nee

the print-based questionnaire

tailored physical activity advice

...with role model stories

Beste mevrouw X,

Welkom bij OncoActief! Voldoende bev[illegible] behandeling voor darmkanker. Wij ond[illegible] beweegadvies, gebaseerd op de antwoo[illegible] beweging wordt aangeraden voor een [illegible] andere) vragen voor u persoonlijk. U [illegible] onze website: www.oncoactief.nl. Lo[illegible] dan contact met ons op!

Norm Gezond Bewegen
Hoeveel beweging is goed voor uw gezondheid? En hoeveel [illegible] voor darmkanker? Minstens 5 dagen per week minimaal een half uur per [illegible] bewegen. Dat is de Norm Gezond Bewegen, ook na de behandeling voo[illegible]anker. Bij [illegible] intensief bewegen versnelt uw ademhaling. U kunt nog gewoon blijve[illegible]n en hoeft niet buiten adem te raken. Bewegen is niet alleen sporten maar ook stevig wande[illegible], fietsen, tuinieren en huishoudelijk werk. Door de behandelingen voor darmkanker kunn[illegible] uw spieren en conditie verzwakt zijn. Bouw het bewegen daarom rustig op. En bedenk dat u door te bewegen werkt aan uw conditie en u uw spieren versterkt, zodat u uiteindelijk ook aan de norm kunt voldoen.

Hoeveel beweegt u?
In onderstaande grafiek ziet u hoeveel minuten u gemiddeld per dag beweegt. De tweede balk geeft aan hoeveel uw vrouwelijke leeftijdsgenoten bewegen: gemiddeld 47 minuten per dag. De derde laat de Norm Gezond Bewegen zien. U heeft aangegeven dat u zelf denkt dat u evenveel beweegt als uw leeftijdsgenoten. Uit de grafiek blijkt echter dat u minder beweegt dan uw leeftijdsgenoten. Dit kan natuurlijk komen doordat u nog aan het herstellen bent van kanker. Maar juist na kanker is het belangrijk om voldoende te bewegen, dus misschien is het toch een goede reden voor u om nog wat meer te gaan bewegen?

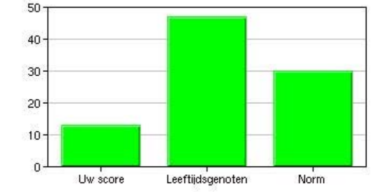

Uit onze berekening blijkt dat u gemiddeld 13 minuten per dag (matig) intensief beweegt. U beweegt dus nog niet de aanbevolen 30 minuten per dag.
In de vragenlijst hebt u ook aangegeven 7 dagen per week, minimaal 30 minuten per dag bezig te

ONCO actief

longaandoening. Overleg met uw arts wanneer u twijfelt of een bepaalde activiteit voor u wel geschikt is.

[illegible]ordelen van bewegen
[illegible] de vragenlijst gaf u aan al vele voordelen van voldoende bewegen te zien. Dat is goed! Want [illegible]oldoende bewegen is ook erg prettig, gezellig, ontspannend en kan u een voldaan gevoel geven!

[illegible]Matty en Valentin (voormalige kankerpatiënten) vertellen hieronder over de voordelen van bewegen die zij ervaren hebben.

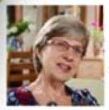

Matty
"Ik heb vaker van artsen gehoord dat het fijn was dat ik een redelijk goede conditie had. Dat maakte de kans om door een operatie heen te komen, en de kans om eerder te herstellen, groter. Als je weet dat dat helpt om beter te worden, om je beter te voelen, dan is het eigenlijk niet moeilijk om te gaan bewegen en dan te zien wat je kunt."

Valentin
"Als je beweegt, en of dat nu wandelen, hardlopen of fietsen is, dan kun je je geest rein maken, op andere gedachten komen, de dagelijkse sleur vergeten. En je komt dan in een omgeving die je altijd weer inspireert om weer andere dingen te zien."

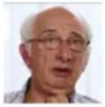

Maar blijkbaar hebben deze redenen u niet kunne[illegible] overtuigen. Daarom zetten wij hi[illegible] enkele goede redenen op een rijtje:

- *U heeft meer energie en minder last van ver[illegible]* Door [illegible] uithoudingsvermogen waardoor u meer energie[illegible] actiever zijn. Bovendien verbetert beweging oo[illegible] kunt beginnen.
- *U krijgt een betere controle* [illegible] betere controle over [illegible] bekkenbodemthe[illegible] versterken.
- *U verkleint de ka*[illegible] voldoende bewege[illegible] belangrijk om voldo[illegible]
- *U krijgt meer sociale* [illegible]wandeling dan tijdens het tv kijken[illegible] te bewegen.
- *U boekt vooruitgang.* U zu[illegible] beweegt, u zich al snel fitter gaat voelen waardoor beweg[illegible]r en leuker wordt.

Bewegen kan u helpen bij het verder gaan met uw leven na kanker. Over het algemeen hebben mensen die voldoende bewegen een betere kwaliteit van leven!
In de vragenlijst noemde u ook een minpunt van meer bewegen:

Beweegplan

Vanaf ga ik meer bewegen!

Denk aan:
- beweegpartner regelen
- geschikte kleding en schoenen
- is alles wat ik nodig heb klaar voor gebruik?
- een beloning voor mij zelf:

	Maandag	Dinsdag	Woensdag	Donderdag	Vrijdag	Zaterdag	Zondag
**Ochtend**	*Tijd: tot*<br>*Wat:*<br>*Waar:*<br>*Met wie:*						
**Middag**							
**Avond**							

physical activity plans

ONCO actief

Oefening 1

Deze eerste oefening versterkt uw kuiten en enkels en verbetert uw stabiliteit en balans.

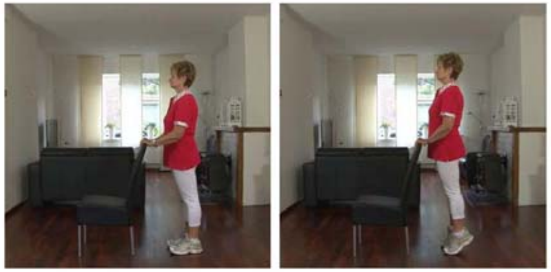

- Ga met uw voeten op schouderbreedte achter een stoel staan, of een achter een ander voorwerp dat u eventueel houvast kan bieden.
- Ga op uw tenen staan en probeer uw hakken *hierbij zo hoog mogelijk te houden.*
- Houd deze positie 2 tot 4 seconden vast, en beweeg vervolgens uw hakken weer langzaam naar de vloer.
- Gebruik de stoel tijdens deze oefening niet om op te leunen, maar gebruik deze alleen wanneer u deze nodig heeft om uw balans te vinden.
- Herhaal deze oefening 10 keer.
- Neem vervolgens 1 minuut pauze, en herha[illegible] de oefening dan nogmaals 10 keer.

exercises to do at home

Hoeveel beweegt u nu?			
	Aantal stappen op de stappenteller	Extra stappen 10 min = 1500 stappen	Totaal aantal stappen
maandag			
dinsdag			
woensdag			
donderdag			
vrijdag			
zaterdag			
zondag			
Gemiddeld aantal stappen per dag =			

instructions to set pedometer goals

Week 1: Mijn doel: ...... stapp[illegible] per dag			
	Aantal stappen op de stappenteller	Extra stappen 10 min = 1500 stappen	Totaal aantal stappen
maandag			
dinsdag			
woensdag			
donderdag			
vrijdag			
zaterdag			
zondag			
Gemiddeld aantal stappen per dag =			

OncoActive

… is an easy accessible PA intervention for prostate and colorectal cancer patients

… has the potentional to reach a large group of patients against low costs

… RCT results will provide insight into effectiveness

More information

Rianne Golsteijn
Open University of the Netherlands

Rianne.Golsteijn@ou.nl
